# Gaucher disease: single gene molecular characterization of one-hundred Indian patients reveals novel variants and the most prevalent mutation

**DOI:** 10.1186/s12881-019-0759-1

**Published:** 2019-02-14

**Authors:** Jayesh Sheth, Riddhi Bhavsar, Mehul Mistri, Dhairya Pancholi, Ashish Bavdekar, Ashwin Dalal, Prajnya Ranganath, Katta M Girisha, Anju Shukla, Shubha Phadke, Ratna Puri, Inusha Panigrahi, Anupriya Kaur, Mamta Muranjan, Manisha Goyal, Radha Ramadevi, Raju Shah, Sheela Nampoothiri, Sumita Danda, Chaitanya Datar, Seema Kapoor, Seema Bhatwadekar, Frenny Sheth

**Affiliations:** 1 FRIGE’s Institute of Human Genetics, FRIGE House, Jodhpur Gam Road, Satellite, Ahmedabad, Gujarat 380015 India; 2 King Edward Memorial Hospital, Pune, 411011 India; 3 Centre for DNA Fingerprinting and Diagnostics, Hyderabad, 500039 India; 4 Kasturba Medical College, Tiger Cir Rd, Madhav Nagar, Manipal, 576104 Karnataka India; 5 Sanjay Gandhi Postgraduate Institute of Medical Sciences, Lucknow, 226014 India; 6 Sir Ganga Ram Hospital, New Delhi, 110060 India; 7 The Postgraduate Institute of Medical Education and Research, Chandigarh, 160012 India; 8 King Edward Memorial Hospital, Mumbai, 400012 India; 9 J.K. Lone Mother and Child Hospital, Jaipur, 302004 India; 10 Rainbow Children’s Hospital, Telangana, 500034 India; 11 Ankur Institute of Child Health, Ahmedabad, 380009 India; 12 Amrita Institute of Medical Sciences & Research Centre, Cochin, 682041 India; 13 Christian Medical College & Hospital, Vellore, 632004 India; 14 Sahyadri Medical Genetics & Tissue Engineering Facility, Pune, 411038 India; 15 Maulana Azad medical College and Associated Loknayak Hospital, New Delhi, 110002 India; 16 Sterling Hospital, Ahmedabad, 380052 India

**Keywords:** Gaucher disease, β-Glucosidase, Chitotriosidase, *GBA1* gene, Glucocerebrosidase, Indian population, p.Leu483Pro most common mutation, novel mutations in *GBA1* gene

## Abstract

**Background:**

Gaucher disease is a rare pan-ethnic, lysosomal storage disorder resulting due to beta-Glucosidase (*GBA1*) gene defect. This leads to the glucocerebrosidase enzyme deficiency and an increased accumulation of undegraded glycolipid glucocerebroside inside the cells’ lysosomes. To date, nearly 460 mutations have been described in the *GBA1* gene. With the aim to determine mutations spectrum and molecular pathology of Gaucher disease in India, the present study investigated one hundred unrelated patients (age range: 1 day to 31 years) having splenomegaly, with or without hepatomegaly, cytopenia and bone abnormality in some of the patients.

**Methods:**

The biochemical investigation for the plasma chitotriosidase enzyme activity and β-Glucosidase enzyme activity confirmed the Gaucher disease. The mutations were identified by screening the patients’ whole *GBA* gene coding region using bidirectional Sanger sequencing.

**Results:**

The biochemical analysis revealed a significant reduction in the β-Glucosidase activity in all patients. Sanger sequencing established 71 patients with homozygous mutation and 22 patients with compound heterozygous mutation in *GBA1* gene. Lack of identification of mutations in three patients suggests the possibility of either large deletion/duplication or deep intronic variations in the *GBA1* gene. In four cases, where the proband died due to confirmed Gaucher disease, the parents were found to be a carrier. Overall, the study identified 33 mutations in 100 patients that also covers four missense mutations (p.Ser136Leu, p.Leu279Val, p.Gly383Asp, p.Gly399Arg) not previously reported in Gaucher disease patients. The mutation p.Leu483Pro was identified as the most commonly occurring Gaucher disease mutation in the study (62% patients). The second common mutations identified were p.Arg535Cys (7% patients) and RecNcil (7% patients). Another complex mutation Complex C was identified in a compound heterozygous status (3% patients). The homology modeling of the novel mutations suggested the destabilization of the GBA protein structure due to conformational changes.

**Conclusions:**

The study reports four novel and 29 known mutations identified in the *GBA1* gene in one-hundred Gaucher patients. The given study establishes p.Leu483Pro as the most prevalent mutation in the Indian patients with type 1 Gaucher disease that provide new insight into the molecular basis of Gaucher Disease in India.

**Electronic supplementary material:**

The online version of this article (10.1186/s12881-019-0759-1) contains supplementary material, which is available to authorized users.

## Background

Gaucher disease (GD), an autosomal recessively inherited disease, is caused due to a defect in glucosylceramidase beta gene (*GBA1*; OMIM*606463) coding for the enzyme glucocerebrosidase (EC 3.2.1.45; alternate names: acid β-glucosidase and GCase). Its prevalence is 1:50000 in general population and 1:950 live births in Ashkenazi Jewish population [1, 2].

Since Philippe Charles Ernest Gaucher first described the GD phenotypes a hundred and thirty-six years ago, the eponym ‘Gaucher Disease’ was introduced. In 1934, a study by Aghion revealed that the distorted macrophages, also called Gaucher cells, resulted due to an accumulation of the lipid glucocerebroside [3]. In 1965, a study by Dr. Brady and Dr. Patrick uncovered the fact that the metabolic defect in GD was due to deficiency of the enzyme β-Glucocerebrosidase [4, 5]. In 1968, the GD was classified as a Lysosomal storage disorder since the Weinreb et.al. work on a rat model established the Lysosomal localization of β-Glucocerebrosidase [6].

The multi-systemic GD has heterogeneous phenotypes, however, based on the age of onset, presence/absence and progression of neurologic manifestation; the GD is clinically divided into three types. GD type 1 (non-neurological) is the most common form whereas neuronopathic GD i.e. type 2 (acute neuronopathic) and type 3 (chronic neuronopathic) occurs less frequently. The neuronal involvement in the GD is a grey area. Though, a study by Kinghorn et al. on an orthologs *GBA1* gene knocked out Drosophila model, demonstrated that the blocking of autophagy flux results in the glucosylceramide accumulation in the brain and age-dependent locomotor defects [7]. The most common clinical features in GD include hepatosplenomegaly, anemia, thrombocytopenia, growth retardation, seizures, and radiological bone disease.

The loss-of-function mutations in the *GBA1* gene prevent the GCase enzyme from cleaving the β-glucosyl linkage of Glucocerebroside, required to break down glycolipid glucocerebroside into glucose and ceramide [8]. The human *GBA1* gene (12 exons and 11 introns), located on chromosome 1q22, has a highly homologous pseudogene sequence located 16 kb downstream which shares 96% exonic homology with the functional gene [9]. As per the Human Genome Mutation Database (HGMD), nearly 460 disease causing *GBA1* mutations have been reported in GD. The first mutation reported in GD was p.Leu483Pro [10]. The most common mutation identified in the Romanian patients is p.Asn409Ser [11]. These mutations along with c.84dupG (84GG) and IVS2 + 1G>A accounts for the 96% of mutations in Ashkenazi Jew patients and approximately 50–60% of mutations in non-Jewish Gaucher’s disease patients [12, 13]. Our recent publication established that the carrier frequency of p.Leu483Pro in Indian population is 1:600 [14].

The given study intended to explore the molecular features of GD in Indian patients. Hence, 100 Indian Gaucher patients were screened by DNA sequencing of the *GBA1* gene to identify the unknown and common Indian mutations for GD and understand the genotypic effect on the phenotype of these patients. The study also aimed at developing a common molecular screening test for GD in India.

## Methods

### Patients

The present study comprises the patients referred as clinical cases from Institute of Human Genetics after genetic counselling as well as from outside referring physicians. The Ethics committee of the Foundation for Research in Genetics and Endocrinology (FRIGE) at the Institute of Human Genetics approved the study and it was performed in accordance with the tenets of the Declaration of Helsinki. Irrespective of the case reference, a written informed consent for investigation and publication of the data was obtained from the patients or their guardian as per the institutional ethics committee guidelines.

The present study on GD was carried out in 100 unrelated patients (90 children, 8 adults, and 2 fetal tissues) referred in the time from 2011 to 2018 with a clinical suspicion of GD. The patients’ common clinical presentation included mild to severe liver/spleen enlargement, anaemia, thrombocytopenia and presence of Gaucher cells in bone marrow.

### Biochemical investigations

The plasma, leukocyte, and genomic DNA (gDNA) was isolated from each patient using six millilitres of blood, drawn in ethylenediaminetetraacetic acid vacutainer.

### Plasma chitotriosidase screening

A previously described protocol was followed for the estimation of chitotriosidase enzyme activity in blood plasma [15]. In brief, the plasma along with the substrate 4-MU (4-Methylumbelliferyl-β-D-N,N′,N″-triacetylchitotrioside) were incubated at 37 °C and the fluorescence was measured using photo fluorometer (360 nm primary and 465 nm secondary filter).

### β-Glucosidase enzyme assay

The substrate 4-methylumbelliferyl-β-D-glucopyranoside was used to measure the Lysosomal hydrolase enzyme (β-Glucosidase) activity from leukocyte using fluorometric assay [16].

### Molecular genetics investigations

#### DNA extraction and purification

DNA, isolated from whole blood using the standard salting-out method, was quantified using a QIAxpert (Cat. No: 9002340) from Qiagen [17]. The DNA samples were purified using The Genomic DNA Clean & Concentrator™-25 (DCC™) Kit, from Zymo Research, Irvine, California, U.S.A (Cat. No. D4064) and were stored at − 20 °C until investigated.

### Primary screening of the common Gaucher mutations (p.Leu483Pro)

Our earlier Polymerase Chain Reaction- Restriction Fragment Length Polymorphism (PCR-RFLP) based protocol for p.Leu483Pro screening was applied [18]. The Thermal Cycler-2720 (Applied Biosystems, Inc. India) amplified the DNA samples and the restriction endonucleases *MspI* (New England Biolabs) digested the PCR product. In brief, 10 μl of PCR product along with 0.5 μL of *MspI* (10 U/μl) enzyme was incubated at 37 °C for 3 h. The digested DNA fragments were separated on 2.5% agarose gel.

The above protocol also covers the mutation p.Arg463Cys that can be distinguished from p.Leu483Pro based on the different band pattern of the DNA fragments on the agarose gel. The codon numbering of the mutations described in this study uses the current nomenclature for *GBA1* mutations that includes the first 39 amino acids of the leader sequence.

### Single-gene sequencing (*GBA1* gene)

The *GBA1* gene containing 12 exons was amplified using primer sets standardized by nested PCR for specific amplification of the functional gene (see Additional file 1). For exons 1–3, 35 cycles of amplification; each consisting of initial denaturation (94 °C; 4 min), denaturation (94 °C; 30 s), annealing (65.5 °C; 30 s), elongation (72 °C; 30 s), and final elongation (72 °C; 10 min) were run. Amplification for exon 4–5 involved initial denaturation (96 °C; 2 min), denaturation (96 °C; 30 s), annealing (61 °C; 30 s), elongation (74 °C; 60 s), and final elongation (74 °C; 5 min) were run. Exon 6–12 included initial denaturation (96 °C; 2 min) followed by 33 cycles each consisting of denaturation (96 °C; 30 s), annealing (58 °C to 61 °C; 30 s), elongation (74 °C; 60 s), and final elongation (74 °C; 5 min).

The *GBA1* gene Sanger sequencing was performed on the Applied Biosystems™ SeqStudio™ Genetic Analyzer with SeqStudio™ Data Collection Software using our earlier described protocol [14]. In brief the cycle sequencing products (samples) were purified, resuspended in Hi-Di Formamide and transferred to 96 wells plate, where the samples were denatured at 95 °C for 2 min and snap chilled at -20 °C for 2–3 min before proceeding with the sequencing. The sequences obtained were aligned to the available reference sequence (NM_001005741.2) in The National Centre for Biotechnology Information (NCBI) GeneBank database to detect variation.

### In Silico analysis

#### Prediction of the functional effect of the variants

Six in silico tools identifying the effect of DNA mutations (MutationTaster2), coding non-synonymous variants (SIFT), coding and non-coding variants (FATHMM), and amino acid substitution (PolyPhen2, PROVEAN, and Mutation Assessor) were employed.

### Orthologous conservation of the GBA residues harbouring the novel variant

A previously described protocol to check the inter-species conservation of the GBA residues incorporating novel variants was employed [19]. In brief, the Clastal Omega; an online multiple sequence alignment program was used to align the *Homo sapiens* (NP_001005741) protein sequence along other species.

### Homology modeling, structure validation and protein stability due to novel variants

The mutated protein structure of the four novel missense variants was modeled using our previously described protocol [19]. In brief, the mutant protein model was build using the template β-Glucosidase crystallographic structure (PDB ID: 1OGS) and the Root Mean Square Deviation (RMSD) of the mutant structures with respect to the wild-type structure was calculated.

## Results

The patients included in the study were in the age range 1 day to 31 years at the time of investigations. The study comprises 62 male and 36 female patients presented with unexplained hepatomegaly, moderate splenomegaly, anaemia, and thrombocytopenia with or without any bone abnormality. Bone marrow examination in the majority of the patients showed classical Gaucher cells. Table 1 covers the demographic profile of the patients. Out of 100 patients investigated, 75 patients were diagnosed with GD type I, 12 patients were diagnosed with GD type II, and 11 patients were diagnosed with GD type III. Two fetuses were diagnosed with GD by enzymatic study however, their GD type remains indistinct. Parental consanguinity was observed in 26 (26%) patients. The parents in consanguineous marriage were first-degree relatives and hence the average inbreeding coefficient of the 26 patients is 3.1%. Majority of the patients were observed from the western India (43%; Gujarat and Maharashtra), followed by the northern zone (32%; Rajasthan, Punjab, New Delhi, Himachal Pradesh, Jammu and Kashmir, and Uttar Pradesh), southern zone (21%; Karnataka, Kerala, Telangana, and Tamil Nadu), and eastern zone (4%; Bihar, and West Bengal).

**Table 1:** Demographic profile of the patients with Gaucher disease

	Total patients *n* = 100	GDI *n* = 77	GDII *n* = 12	GDIII *n* = 11
Gender				
Male	62 (62%)	47 (61.03%)	7 (58.33%)	8 (72.72%)
Female	36 (36%)	28 (36.36%)	5 (41.66%)	3 (27.27%)
Fetus	2 (2%)	–	–	–
Regional distribution				
East India	4 (4%)	3 (3.89%)	1 (8.33%)	–
West India	43 (43%)	32 (41.55%)	7 (58.33%)	4 (36.36%)
North India	32 (32%)	24 (31.16%)	3 (25%)	5 (45.45%)
South India	21 (21%)	18 (23.37%)	1 (8.33%)	2 (18.18%)

Abbreviations: *GDI*, Gaucher disease type I, *GDII* Gaucher disease type II, *GDIII* Gaucher disease type III

Data are represented as n (%)

### Biochemical analysis

Out of 100 patients, plasma chitotriosidase was elevated (172–72,000 nmolh^−1^ml^−1^plasma) in 71 patients, undetectable in 5 patients, and normal (1.0–102.4 nmolh^−1^ml^−1^plasma) in 13 patients. However, no information about the plasma chitotriosidase activity in 11 patients is available. The β-Glucosidase enzyme assay confirmed the GD. Significantly reduced activity (< 10% of normal mean) of the β-Glucosidase enzyme was observed in the patients (see Additional file 2). The β-Glucosidase enzyme activity results were not available in five patients.

### Molecular analysis

**Fig. 1:**
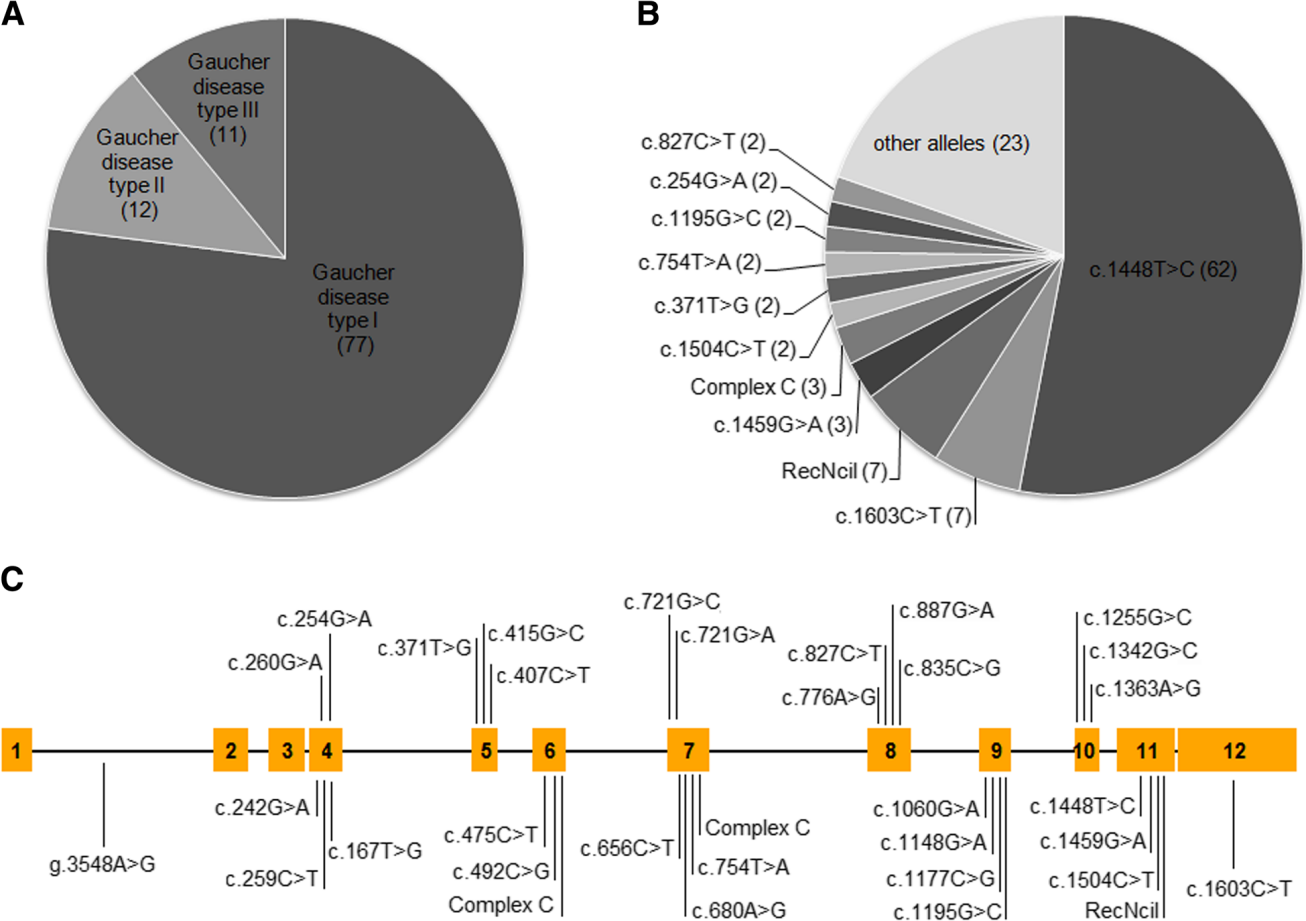
Illustrative representation of the distributions of the variants identified in Indian Gaucher patients investigated in this study. **a** Majority of the patients were affected with Gaucher disease type I (77 patients), followed by Gaucher disease type II (12 patients) and Gaucher disease type III (11 patients). **b** The most common mutation c.1448T>C was observed in 62 patients (including homozygotes and compound heterozygotes). The second most common mutations identified were c.1603C>T and RecNcil. **c** Variations on exon 4–12 were observed on *GBA1* gene. Also a mutation g.3548A>G was observed in the intron 1. The mutations were more clustered on exon 4 and exon 7 to exon 11

The bidirectional sequencing of the coding *GBA1* gene revealed a total 33 mutations in 93 patients. Figure 1 depicts an illustrative representation of the variants identified through Sanger sequencing.

The identification of the genetic cause of GD in all the patients involved an initial screening for the common mutations c.1448 T>C (p.Leu483Pro) observed in Indian patients with GD [18, 20]. Patient P_1_ to P_51_ were found to be homozygous for p.Leu483Pro mutation in exon 11 of *GBA1* gene (Table 2). Patient P_52_ to P_62_ harbour a heterozygous copy of the same mutation with the second disease-causing mutant allele. Hence, the mutation p.Leu483Pro was observed as a most common mutation in the patients affected with GD (including homozygous and compound heterozygous). However, during the initial screening, no molecular output was obtained in the remaining patients. As a single variant or no variant was detected in the initial screening, the further investigation involved the sequencing of the complete coding region of *GBA1* gene in these patients.

**Table 2:** Molecular analysis of the patients with Gaucher disease

Patient ID	Variant location (*GBA1* gene^b^)	Zygosity	Allele frequency		dbSNP reference number
			1000 Genomes	ExAC	
P_1_-P_51_	Ex11:c.1448T>C/p.L483P	Hom	0.0034	0.0031	rs421016
P_52_	Ex11:c.1448T>C/p.L483P	Com Hetz	0.0034	0.0031	rs421016
	Ex5:c.407C>T/p.S136 L^a^		NR	NR	rs878853316
P_53_	Ex11:c.1448T>C/p.L483P	Com Hetz	0.0034	0.0031	rs421016
	Ex4:c.167T>G/p.V56G		NR	NR	rs878853318
P_54_	Ex11:c.1448T>C/p.L483P	Com Hetz	0.0034	0.0031	rs421016
	Ex10:c.1363A>G/p.M455 V		NR	NR	NR
P_55_	Ex11:c.1448T>C/p.L483P	Com Hetz	0.0034	0.0031	rs421016
	Ex7:c.656C>T/p.T219I		NR	NR	NR
P_56_-P_57_	Ex11:c.1448T>C/p.L483P	Com Hetz	0.0034	0.0031	rs421016
	Ex12:c.1603C>T/p.R535C		NR	0.00004357	rs747506979
P_58_-P_59_	Ex11:c.1448T>C/p.L483P	Com Hetz	0.0034	0.0031	rs421016
	Ex5:c.371T>G/p.M124R		NR	0.000008237	NR
P_60_	Ex11:c.1448T>C/p.L483P	Com Hetz	0.0034	0.0031	rs421016
	Ex10:c.1255G>C/p.D419H		NR	NR	NR
P_61_-P_62_	Ex11:c.1448T>C/p.L483P	Com Hetz	0.0034	0.0031	rs421016
	Ex11:RecNcil		–		
P_63_-P_64_	Ex12:c.1603C>T/p.R535C	Hom	NR	0.00004357	rs747506979
P_65_	Ex12:c.1603C>T/p.R535C	Com Hetz	NR	0.00004357	rs747506979
	Ex6,7:Complex C		–		
P_66_-P_67_	Ex11:c.1504C>T/p.R502C	Hom	NR	0.00006	rs80356771
P_68_-P_70_	Ex11:c.1459G>A/p.A487T	Hom	NR	NR	rs878853317
P_71_	Ex6:c.492C>G/p.S164R	Com Hetz	NR	0.00003295	NR
	Ex4:c.254G>A/p.G85E		NR	0.000008	rs77829017
P_72_	Ex4:c.254G>A/p.G85E	Com Hetz	NR	0.000008	rs77829017
	Ex11:RecNcil		–		
P_73_-P_74_	Ex7:c.754T>A/p.F252I	Hom	NR	0.00002	rs381737
P_75_-P_76_	Ex9:c.1195G>C/p.G399R^a^	Com Hetz	NR	NR	NR
	Ex12:c.1603C>T/ p.R535C		NR	0.00004357	rs747506979
P_77_	Ex7:c.721G>A/p.G241R	Hom	NR	NR	rs409652
P_78_	Ex10:c.1342G>C/p.D448H	Hom	NR	0.0001	rs1064651
P_79−_P_80_	Ex8:c.827C>T/p.S276F	Hom	NR	0.00000837	NR
P_81_	Ex9:c.1060G>A/p.D354N	Hom	NR	0.000008	rs398123526
P_82_	Ex8:c.776A>G/p.Y259C	Hom	NR	NR	NR
P_83_	Ex5:c.415G>C/p.A139P	Hom	NR	NR	rs878853314
P_84_	Ex9:c.1177C>G/p.L393 V	Hom	NR	NR	rs878853315
P_85_	Ex7:c.721G>C/p.G241R	Hom	NR	NR	NR
P_86_	Ex4:c.260G>A/p.R87Q	Hom	NR	NR	rs78769774
P_87_	Ex8:c.835C>G/p.L279 V^†^	Hom	NR	NR	NR
P_88_	In1:g.3548A>G/g.3548A > G	Com Hetz	0.0078	NR	rs18897815
	Ex11:RecNcil		–		
P_89_	Ex4:c.259C>T/p.R87W	Com Hetz	NR	0.00002	rs1141814
	Ex11:RecNcil		–		
P_90_	Ex9:c.1148G>A/p.G383D^†^	Com Hetz	NR	NR	NR
	Ex11:RecNcil		–		
P_91_	Ex6:c.475C>T/p.R159W	Com Hetz	NR	NR	rs439898
	Ex11:RecNcil		–		
P_92_	Ex7:c.680A>G/p.N227S	Com Hetz	0.0002	0.00007	rs364897
	Ex6,7:Complex C		–		
P_93_	Ex8:c.887G>A/p.R296Q	Com Hetz	NR	0.00003	rs78973108
	Ex6,7:Complex C		–		
P_94_	Patients’DNA is not available Mother- Ex11:c.1448T> C/p.L483P Father- Ex11:c.1448T> C/p.L483P	Single Hetz	0.0034	0.0031	rs421016
P_95_	Patients’ DNA is not available Mother- Ex11:c.1448T>C/p.L483P	Single Hetz	0.0034	0.0031	rs421016
	Father- Ex4:c.242G>A/p.S81 N		NR	NR	NR
P_96_	Patients’ DNA is not available Mother- Ex6:c.475C>T/p.R159W	Single Hetz	NR	NR	rs439898
	Father- Ex11:c.1448T>C/p.L483P		0.0034	0.0031	rs421016
P_97_	Patients’ DNA is not available Mother- Ex6,7:Complex C Father- Ex6,7:Complex C	Single Hetz	–		
P_98_	Mutation not found	NA	NA	NA	NA
P_99_	Mutation not found	NA	NA	NA	NA
P_100_	No amplification from Exon 1 to Exon 7	NA	NA	NA	NA

Abbreviations: *Com Hetz* Compound Heterozygous, *dbSNP* The Single Nucleotide Polymorphism database, *ExAC* The Exome Aggregation Consortium, *Ex* Exon, Homozygous (Hom), *NR* Not Reported

RecNcil: [c.1448T>C (p.L483P), c.1483G>C (p.A495P), c.1497G>C (p.V499 V)]]

Complex C: [c.475C>T (p.R159W), c.667T>C (p. W223R), c.681T>G (p.N227K), c.689 T>G (p.V230G), c.703T>C (p.S235P), c.721G>A (p.G241R), c.754T>A (p.F252I)]

^a^Novel variants identified in the given study

^b^The above variants refers to the *GBA1* gene with transcript ID ENST00000327247.5 and reference sequence number NM_001005741.2

The second most common mutations identified in the given study are c.1603C>T (p.Arg535Cys) in exon 12 and the complex mutation RecNcil [c.1448 T>C (p.L483P), c.1483G>C (p.A495P), c.1497G>C (p.V499 V)] in exon 11. These mutations existed in both homozygous and compound heterozygous status in seven patients each. Another complex mutation Complex C [c.475C>T (p.R159W), c.667T>C (p.W223R), c.681T>G (p.N227K), c.689T>G (p.V230G), c.703T>C (p.S235P), c.721G>A (p.G241R), c.754T>A (p.F252I)] was identified in a compound heterozygous status in three patients. Also, the mutation c.1459G>A (p.Ala487Thr) was revealed in three patients in homozygous form.

The given study uncovered the known mutations like c.1504C>T (p.Arg502Cys), c.371T>G (p.Met124Arg), c.754T>A (p.Phe252Ile), c.827C>T (p.Ser276Phe), and c.254G>A (p.Gly85Glu); each in different sets of two patients. These mutations were comparatively less commonly observed.

**Fig. 2:**
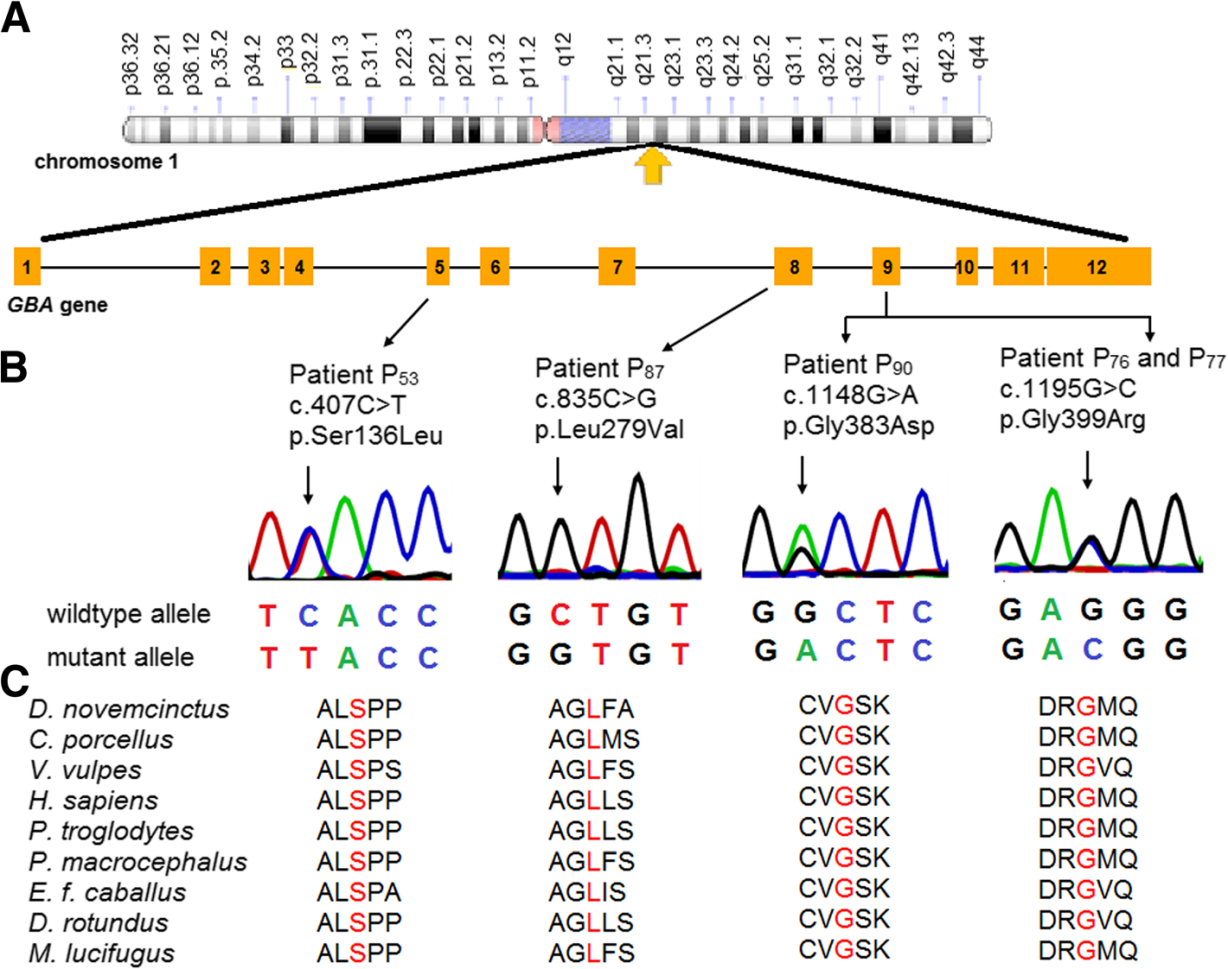
Identification of novel variants in *GBA1* gene. **a** Illustrative representation of the distributions of the novel variants identified in Indian Gaucher patients investigated in this study. **b** Sanger sequencing discovered four missense variants in *GBA1* gene. The variants p.Ser136Leu, p.Gly383Asp, and p.Gly399Arg, were identified, as compound heterozygotes along with another know mutant allele. The variant p.Leu279Val existed in homozygous form. An arrow indicates the point of variation. **c** The multiple alignment of the protein sequence surrounding the novel variants against various orthologous sequence revealed the conservative status of the wildtype residues (marked red)

The sequencing of the coding region of *GBA1* gene revealed four novel variants in the given study (Fig. 2a and b). The novel variant c.835C>G (p.Leu279Val) was identified in the homozygous state in exon 8. The remaining three novel variants, c.407C>T (p.Ser136Leu), c.1195G>C (p.Gly399Arg), and c.1148G>A (p.Gly383Asp) were identified as compound heterozygous along with other known mutations (Table 2).

Apart from this, the study also found 18 known mutations; each in one patient. These mutations are g.3548A>G, c.167T>G (p.Val56Gly), c.1363A>G (p.Met455Val), c.656C>T (p.Thr219Ile), c.1255G>C (p.Asp419His), c.492C>G (p.Ser164Arg), c.721G>A (p.Gly241Arg), c.1342G>C (p.Asp448His), c.1060G>A (p.Asp354Asn), c.776A>G (p.Tyr259Cys), c.415G>C (p.Ala139Pro), c.1177C>G (p.Leu393Val), c.721G>C (p.Gly241Arg), c.260G>A (p.Arg87Gln), c.259C>T (p.Arg87Trp), c.475C>T (p.Arg159Trp), c.680A>G (p.Asn227Ser), c.887G>A (p.Arg296Gln).

The carrier analysis was carried out in four couples where the proband died due to confirmed GD. In case of the patient P_94_, the parents were found to be the carrier for the most commonly identified mutation p.Leu483Pro. While one parent in case of patients P_95_ and P_96_ was heterozygous for p.Leu483Pro. The following compound heterozygous mutation identified in the second parent of the patient P_95_ and P_96_ were c.242G>A (p.Ser81Asn) and c.475C>T (p.Arg159Trp) respectively. For the patient P_97_, the parents were identified as a carrier for the Complex C.

However, no variation in the exonic or exon-intronic boundaries of *GBA1* gene was detected in three patients (P_98_, P_99_, and P_100_). These patients presented common clinical indications like hepatosplenomegaly, unexplained hepatomegaly, moderate splenomegaly, anemia, and thrombocytopenia with or without any bone abnormality as also indicated in GD patients. Along with these common clinical indications, patient P_99_ and P_100_ manifested neurological regression evident at the age of 8 years and one and half years respectively. Hence, patient P_99_ and P_100_ were classified as GD type 3 and GD type 2 respectively. Their significant decrease in the β-Glucosidase enzyme activity confirmed the GD diagnosis. In such cases of the uninformative Sanger sequencing results, the possibility of either large deletion/duplication or deep intronic variations cannot be ruled out.

Overall, the mutation p.Leu483Pro was observed as a most common mutation with its prevalence in 62% patients affected with GD. Its frequency was 60.75% of the total mutations detected.

### In silico analysis of the novel variants

The in silico tools described above established the functional effects of the variants identified (see Additional file 3). The novel variants were found to be disease causing. These predicting tools suggest the probably damaging and deleterious effect of the novel variants on protein function. These variants were found neither in the 1000 Genomes database nor in the Exome Aggregation Consortium (ExAc).

**Fig. 3:**
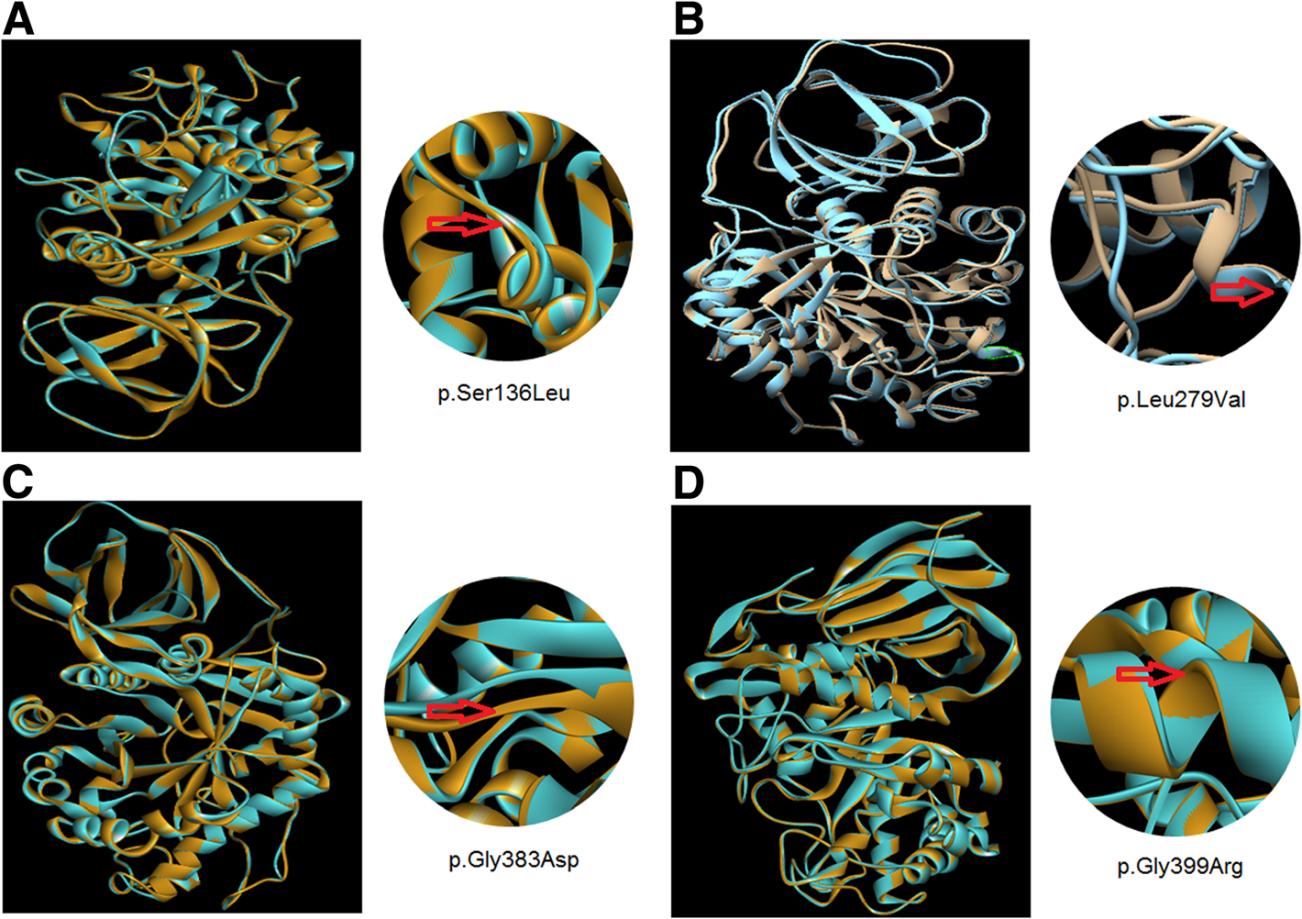
Homology modeling of novel missense variants identified in the *GBA1* gene. The native structure (blue) and mutant structure (brown) are superimposed. **a** The model of the variant p.Ser136Leu depicting the changes in the loop region joining α1 and α1a; at the codon number 136 (TCA-TTA). **b** The model of the variant p.Leu279Val depicting the destabilization of the normal shape of the active site cavity at the codon number 279 (CTG-GTG). **c** The model of the variant p.Gly383Asp depicting the conformational changes in the loop regions at the codon number 383 (GGC-GAC). **d** The model of the variant p.Gly399Arg depicting the conformational changes in the alpha helix at the codon number 399 (GGG-CGG). All the models reveal the conformational changes in the GBA protein structure

The protein sequence alignment of *Homo sapiens* along with other species using Clastal Omega suggests that these variations occurred at highly evolutionarily conserved and functionally active residual domain in the protein (Fig. 2c). The protein homology modeling of the novel missense point variants in the *GBA1* gene suggest their damaging effect at highly evolutionarily conserved and functionally active domain residues in the protein leading to conformational changes or destabilization of the protein structure (Fig. 3). The variant p.Ser136Leu caused changes in the loop region joining α1 and α1a helix. The variant p.Leu279Val caused destabilization of the normal shape of the active site cavity. The variant p.Gly383Asp caused conformational changes in the loop regions and the variant p.Gly399Arg caused conformational changes in the alpha helix.

## Discussion

The given study demonstrates the mutation spectrum of GD in a large Indian cohort. It reveals 100 cases of GD (77 with GD type 1, 12 with GD type 2, and 11 with GD type 3) with maximum GD type 1 cases.

The literature reports p.Leu483Pro, p.Asn409Ser, c.84dupG (84GG) and IVS2 + 1G>A as the most common mutations in GD. A study by Bisariya et al. in 24 Indian GD patients reported the mutant alleles p.Leu483Pro, p.Asn409Ser, IVS2+1G>A, p.Asp448His and c.1263_1317del (55Del) collectively in approximately 50% of the patients; p.Leu483Pro was the most common mutation, followed by p.Asp448His [21]. However, in the given study only p.Leu483Pro was observed as the most common mutation. The other mutations mentioned above were not detected.

Variation in these observations can be likely due to different ethnic group involved in these studies. Endogamous marriage practice has clustered specific community in different zones of India. The majority of the patients, in the given study, are from western and northern zone that provides an idea that GD mutations spectrum may vary in different parts of India. However, the mutation p.Leu483Pro remains as the most commonly observed mutation in Indian GD patients [18, 20, 21]. Considering these results along with our recently published data on carrier frequency of p.Leu483Pro (1:600), it is justifiable to consider p.Leu483Pro as the most prevalent mutation in the Indian population; irrespective of the ethnic group [14].

Several ethnicity specific mutations have been observed in the GD. For instance, the mutation p.Gly416Ser is observed in high frequency in Brazilian GD type 3 patients [22]. The mutations p.Leu483Pro and p.Phe252Ile are relatively common in Japanese GD patients [23, 24]. The most prevalent mutations in Turkish, Romanian, and Czech and Slovak GD patients are p.Leu483Pro and p.Asn409Ser [11, 25, 26]. Based on these, the mutation p.Leu483Pro can be considered Pan-ethnic.

The mutation c.84dupG (84GG), along with other common Jewish GD mutation, accounts for the 50–60% of mutations in non-Jewish GD patients. However, it was observed neither in the given study nor in our previously published data on GD [18]. This may account for the low prevalence of bone disease in the patients investigated [27]. Several studies on non-Jewish population have also failed to identify this mutation [11, 18, 21, 26, 28].

The given study has also identified the complex allele RecNcil in seven patients and Complex C in three patients. These mutations usually result either due to non-homologous recombination between the functional *GBA1* gene and the highly homologous, non-functional *GBA1* pseudogene, gene conversion, fusion or duplication [28, 29]. A study by Horowitz et al. reported RecNcil in 7.8% of alleles of non-Jewish patients [28]. The given study reports two patients (GD type 1 and GD type 2) with p.Leu483Pro/RecNcil. Lee et al. also reported the same mutant complex in a patient with GD type 2 [30]. Nevertheless, the phenotypic heterogeneity prevails amongst these patients.

A study by Koprivica et al. suggests the association of p.Leu483Pro with GD type 3 [31]. However, the given study demonstrates that, though associated with all types of GD, the mutation p.Leu483Pro is most commonly observed in GD type 1. A study on Romanian GD patience was in accordance to our given study [11]. A study by Wan et al. in Taiwanese population have reported the occurrence of p.Leu483Pro in 53.5% of type 1 and type 2 GD patients collectively [32]. Similarly, the p.Leu483Pro mutation was identified in 60% of the total Thai patients with GD type 1 and type 2 [33]. These phenotypic variations in different population, due to p.Leu483Pro mutant allele could be because of the effect of the modifier gene on mutant allele. A Genome-wide Association Study by Zhang et al. identified *CLN8* as a potential modifier gene responsible for the phenotypic variation in the type 1 GD patients with p.Asn409Ser homozygotes [34]. However, the impact of modifier genes on GD is a grey area. It is also likely that many of our type 1 GD patients with p.Leu483Pro genotype may develop neurological symptoms at the later stage as observed in the Spanish population [35]. Owing to the high cost of enzyme replacement therapy and poor financial background of the patients, only two patients with type 1 GD are provided the therapy. However, the long term follows up of our remining patients with type 1 GD will help to understand the heterogenic effect of the said genotype on the phenotype. Those with p.Leu483Pro carriers are also at an increased risk of Parkinson disease (PD) and extended study of these families will help to understand the natural history of progression from carrier to PD. A study by Wang et al. in Chinese population established that patients with p.Leu483Pro had 3.4% chances of developing PD compared to the 0.3% of the controls and hence a long-term follow would be essential to understand the impact of PD on Indian population [36].

The majority of mutations found in the given study were clustered in exons 4, 7, 8, 9, 10, and 11. Around 45% of the mutations have been observed in exons 8 to 11. The expression studies have shown that certain residues substitution in exon 8, 9, 10, and 11 are highly disruptive and can produce compromised protein [37].

## Conclusion

In conclusion, our results confirmed the remarkable heterogeneity of the mutational spectrum of the *GBA1* gene and provided new insight into molecular pathology of GD. The mutation c.1448T>C (p.Leu483Pro) was identified in 62% patients and hence can be used for mass screening. The same mutation was also observed in majority of the patients with GD type I. This is likely to provide the base to understand the progression of some of these patients in type 3 GD and possible effect on the development of Parkinson disease. Since the mutations were clustered in the exons 4, 7, 8, 9, 10, and 11, this region can be considered as hot spot for mutation analysis.

## Additional files

Additional file 1: List of primers used for *GBA1* gene sequencing. The exons and the exon-intron boundaries of both the genes were bidirectionally sequenced using the given set of primers. (DOC 32 kb)

Additional file 2: Biochemical analysis of the Gaucher Disease patients. The plasma chitotriosidase enzyme activity and β-Glucosidase enzyme activity were checked using the standard protocol. (DOCX 17 kb)

Additional file 3: In silico analysis of the functional effect of the variants identified in the patients with Gaucher disease. The in silico tools predicting the effect of DNA variants, amino acid substitution, non-coding variants, and coding non-synonymous variants were employed to predict the functional effect of the variants identified in the given study. (DOCX 20 kb)

Additional file 4: ClinVar Accession ID of the variants generated in the given study. The variants identified through Sanger sequencing are reported in NCBI ClinVar database. The file provides accession ID and the links to an individual variant. (DOCX 14 kb)

## Abbreviations

4-MU: 4-Methylumbelliferyl-β-D-N,N′,N″-triacetylchitotrioside
dbSNP: The Single Nucleotide Polymorphism database
FATHMM: The Functional Analysis Through Hidden Markov Models
*GBA1*: beta-Glucosidase
GD: Gaucher disease
gDNA: genomic DNA
NCBI: National Center for Biotechnology Information
PCR: Polymerase Chain Reaction
PolyPhen-2: Polymorphism Phenotyping version 2
PROVEAN: Protein Variation Effect Analyzer
RFLP: Restriction Fragment Length Polymorphism
RMSD: Root Mean Square Deviation
SIFT: The Sorting Intolerant from Tolerant
