# Supplementary material for: Gaucher disease: single gene molecular characterization of one-hundred Indian patients reveals novel variants and the most prevalent mutation
Source: BMC Med Genet. 2019 Feb 14;20:31. doi: 10.1186/s12881-019-0759-1 (PMC6376752; doi:10.1186/s12881-019-0759-1)
Supplement: Supplementary file 1 — List of primers used for GBA1 gene sequencing. The exons and the exon-intron boundaries of both the genes were bidirectionally sequenced using the given set of primers. (DOC 32 kb) [file 12881_2019_759_MOESM1_ESM.doc]

**Primer sets for *GBA1* gene Sanger sequencing**

| **Location** | **Primers** | |
| --- | --- | --- |
|  | **Sense 5'<---->3'** | **Antisense 3'<---->5'** |
| Exon 1 | GACTTGAGGAAGGGCTCTGA | CAAGAAGGCGCCATTACACT |
| Exon 2 | CCTAAAGTTGTCACCCATAC | CAACCCTTCTGATGACAACT |
| Exon 3 | GGAGAGGGGCTTGCTTTTCA | GGAGGCAGAGGTTGGAATGA |
| Exon 4-5 | CAAGGGGTGAGGAATTTTGA | CACCACTGCACTCCTGTCTC |
| Exon 6-7 | TGGCCCTGACTCAGACACTA | CTGATGGAGTGGGCAAGATT |
| Exon 8 | GGCTGTTCTCGAACTCCTGA | ATAGTTGGGTAGAGAAATCG |
| Exon 9 | AGTTGCATTCTTCCCGTCAC | ATCATGGTTCCCCAGAGTTG |
| Exon 10 | CAGCTGCCTCTCCCACAT | GTGTGCCTCTTCCGAGGTT |
| Exon 11-12 | GAGAGCCAGGGCAGAGCCTC | CTCTTTAGTCACAGACAGCG |
