# Supplementary material for: Gaucher disease: single gene molecular characterization of one-hundred Indian patients reveals novel variants and the most prevalent mutation
Source: BMC Med Genet. 2019 Feb 14;20:31. doi: 10.1186/s12881-019-0759-1 (PMC6376752; doi:10.1186/s12881-019-0759-1)
Supplement: Supplementary file 2 — Biochemical analysis of the Gaucher Disease patients. The plasma chitotriosidase enzyme activity and β-Glucosidase enzyme activity were checked using the standard protocol. (DOCX 17 kb) [file 12881_2019_759_MOESM2_ESM.docx]

Biochemical analysis of the patients with Gaucher Disease

| **Patient ID** | **Plasma Chitotriosidase**  **(nmol/hr/mlplasma)** | **β-Glucosidase**  **(nmol/hr/mg protein)** |
| --- | --- | --- |
| **P_1_** | 4.9 | 1.03 |
| **P_2_** | 3892 | 0.9 |
| **P_3_** | 9227 | NA |
| **P_4_** | NA | NA |
| **P_5_** | 51289 | 4.3 |
| **P_6_** | NA | 2.05 |
| **P_7_** | 28321.6 | 0.33 |
| **P_8_** | 18020.92 | 0.65 |
| **P_9_** | 65980.0 | 1.18 |
| **P_10_** | 10910.0 | 2.0 |
| **P_11_** | NA | NA |
| **P_12_** | 28158.1 | 0.39 |
| **P_13_** | 6.526 | 0.8 |
| **P_14_** | 46789.8 | NA |
| **P_15_** | 70450.3 | 0.49 |
| **P_16_** | 39075.49 | 0.7 |
| **P_17_** | 52135.0 | 1.7 |
| **P_18_** | 54596.0 | 0.5 |
| **P_19_** | 21074.32 | 1.2 |
| **P_20_** | 19378.0 | 0.45 |
| **P_21_** | 42879.0 | 1.7 |
| **P_22_** | 34474.2 | 0.62 |
| **P_23_** | 40253.1 | 2.1 |
| **P_24_** | 992.1 | 1.54 |
| **P_25_** | 0 | 1.45 |
| **P_26_** | 56444.8 | 0.46 |
| **P_27_** | 35021.2 | 0.98 |
| **P_28_** | 23737.1 | 0.7 |
| **P_29_** | 41701.79 | 0.6 |
| **P_30_** | 60442.1 | 0.33 |
| **P_31_** | 59922.01 | 0.608 |
| **P_32_** | 3.4 | 1.5 |
| **P_33_** | 9318.4 | 1.0 |
| **P_34_** | 20609.8 | 0.8 |
| **P_35_** | 211.89 | 2.0 |
| **P_36_** | 19046.4 | 1.30 |
| **P_37_** | 9813.6 | 0.52 |
| **P_38_** | 19236.6 | 0.91 |
| **P_39_** | 21160.0 | 0.83 |
| **P_40_** | 321.76 | 0.66 |
| **P_41_** | 19236.6 | 0.32 |
| **P_42_** | 15603.2 | 0.53 |
| **P_43_** | 19557.2 | 0.51 |
| **P_44_** | 16390.8 | 0.30 |
| **P_45_** | 0.0 | 0.12 |
| **P_46_** | 15764.4 | 0.53 |
| **P_47_** | 6625.9 | 0.70 |
| **P_48_** | 18702.0 | 1.1 |
| **P_49_** | 34452.0 | 3.4 |
| **P_50_** | 47557.1 | 3.4 |
| **P_51_** | 35496.0 | 4.8 |
| **P_52_** | 9938.9 | 0.66 |
| **P_53_** | 1283.9 | 3.59 |
| **P_54_** | 0.2 | 0.3 |
| **P_55_** | NA | 0.63 |
| **P_56_** | 29181 | 1.7 |
| **P_57_** | 5450.4 | 0.4 |
| **P_58_** | 26100.0 | 2.6 |
| **P_59_** | 11776.0 | 0.30 |
| **P_60_** | 0.0 | 0.81 |
| **P_61_** | 228.52 | 1.01 |
| **P_62_** | 21748.1 | 1.0 |
| **P_63_** | 809 | NA |
| **P_64_** | NA | 1.0 |
| **P_65_** | 11755.7 | 1.45 |
| **P_66_** | 7.3 | 2.6 |
| **P_67_** | 28899.5 | 2.36 |
| **P_68_** | 72000 | 1.2 |
| **P_69_** | 0.0 | 1.2 |
| **P_70_** | 102.4 | 4.65 |
| **P_71_** | NA | 0 |
| **P_72_** | 47485.17 | 0.6 |
| **P_73_** | 20.88 | 4.0 |
| **P_74_** | 20.8 | 1.44 |
| **P_75_** | 25853.0 | 1.0 |
| **P_76_** | 15764.4 | 1.4 |
| **P_77_** | 54596.0 | 0.5 |
| **P_78_** | 35964.9 | 0.27 |
| **P_79_** | 3.07 | 0.79 |
| **P_80_** | 31928.3 | 0.5 |
| **P_81_** | 14378.0 | 0.24 |
| **P_82_** | 53.3 | 2.1 |
| **P_83_** | 21.37 | 3.6 |
| **P_84_** | 32.0 | 3.8 |
| **P_85_** | 7308.0 | 3.1 |
| **P_86_** | 22977.0 | 0.38 |
| **P_87_** | 19627.2 | 0.75 |
| **P_88_** | NA | 0.0 |
| **P_89_** | 21908.3 | 1.0 |
| **P_90_** | 8015.2 | 0.7 |
| **P_91_** | NA | 1 |
| **P_92_** | 15940 | 1.2 |
| **P_93_** | NA | Deficient |
| **P_94_** | NA | NA |
| **P_95_** | 1450.27 | 3.38 |
| **P_96_** | NA | 1.07 |
| **P_97_** | NA | Deficient |
| **P_98_** | 30.30 | 0.7 |
| **P_99_** | 40.96 | 1.8 |
| **P_100_** | 171.6 | 0.69 |
