# Supplementary material for: Gaucher disease: single gene molecular characterization of one-hundred Indian patients reveals novel variants and the most prevalent mutation
Source: BMC Med Genet. 2019 Feb 14;20:31. doi: 10.1186/s12881-019-0759-1 (PMC6376752; doi:10.1186/s12881-019-0759-1)
Supplement: Supplementary file 3 — In silico analysis of the functional effect of the variants identified in the patients with Gaucher disease. The in silico tools predicting the effect of DNA variants, amino acid substitution, non-coding variants, and coding non-synonymous variants were employed to predict the functional effect of the variants identified in the given study. (DOCX 20 kb) [file 12881_2019_759_MOESM3_ESM.docx]

***In silico* prediction of the functional effect of the variants identified in the patients with Gaucher disease**

| **Sr. No.** | **Variant location (*GBA1* gene)** | **MutationTaster2** | **SIFT** | **FATHMM** | **PolyPhen-2** | **PROVEAN** | **Mutation Assessor^†^** |
| --- | --- | --- | --- | --- | --- | --- | --- |
| 1 | Ex11:c.1448T>C/p.L483P | Disease causing | Damaging | Damaging | Possibly damaging | Deleterious | Medium |
| 2 | Ex5:c.407C>T/p.S136L | Disease causing | Damaging | Damaging | Possibly damaging | Deleterious | Medium |
| 3 | Ex4:c.167T>G/p.V56G | Disease causing | Damaging | Damaging | Possibly damaging | Deleterious | Medium |
| 4 | Ex10:c.1363A>G/p.M455V | Polymorphism | Damaging | Damaging | Possibly damaging | Deleterious | High |
| 5 | Ex7:c.656C>T/p.T219I | Disease causing | Damaging | Damaging | Possibly damaging | Deleterious | Medium |
| 6 | Ex12:c.1603C>T/p.R535C | Disease causing | Damaging | Damaging | Possibly damaging | Deleterious | Low |
| 7 | Ex5:c.371T>G/p.M124R | Disease causing | Damaging | Damaging | Possibly damaging | Deleterious | Medium |
| 8 | Ex10:c.1255G>C/p.D419H | Disease causing | Damaging | Damaging | Possibly damaging | Deleterious | High |
| 9 | Ex11:c.1504C>T/p.R502C | Disease causing | Damaging | Damaging | Possibly damaging | Deleterious | Medium |
| 10 | Ex11:c.1459G>A/p.A487T | Disease causing | Damaging | Damaging | Possibly damaging | Deleterious | Medium |
| 11 | Ex6:c.492C>G/p.S164R | Disease causing | Damaging | Damaging | Possibly damaging | Deleterious | High |
| 12 | Ex4:c.254G>A/p.G85E | Disease causing | Damaging | Damaging | Possibly damaging | Deleterious | Medium |
| 13 | Ex7:c.754T>A/p.F252I | Disease causing | Damaging | Damaging | Benign | Deleterious | Medium |
| 14 | Ex9:c.1195G>C/p.G399R | Disease causing | Damaging | Damaging | Possibly damaging | Deleterious | Medium |
| 15 | Ex7:c.721G>A/p.G241R | Disease causing | Tolerated | Damaging | Possibly damaging | Deleterious | Medium |
| 16 | Ex10:c.1342G>C/p.D448H | Disease causing | Tolerated | Damaging | Benign | Deleterious | Medium |
| 17 | Ex8:c.827C>T/p.S276F | Disease causing | Damaging | Damaging | Benign | Deleterious | Medium |
| 18 | Ex9:c.1060G>A/p.D354N | Disease causing | Tolerated | Damaging | Probably damaging | Deleterious | Low |
| 19 | Ex8:c.776A>G/p.Y259C | Disease causing | Damaging | Damaging | Possibly damaging | Deleterious | High |
| 20 | Ex5:c.415G>C/p.A139P | Disease causing | Tolerated | Damaging | Possibly damaging | Deleterious | Medium |
| 21 | Ex9:c.1177C>G/p.L393V | Disease causing | Damaging | Damaging | Possibly damaging | Deleterious | Medium |
| 22 | Ex7:c.721G>C/p.G241R | Disease causing | Tolerated | Damaging | Possibly damaging | Deleterious | Medium |
| 23 | Ex4:c.260G>A/p.R87Q | Disease causing | Damaging | Damaging | Possibly damaging | Deleterious | Medium |
| 24 | Ex8:c.835C>G/p.L279V | Disease causing | Tolerated | Damaging | Benign | Neutral | Low |
| 25 | In1:g.3548A>G/g.3548A>G | Polymorphism | NA | Damaging | NA | NA | NA |
| 26 | Ex4:c.259C>T/p.R87W | Disease causing | Damaging | Damaging | Possibly damaging | Deleterious | Medium |
| 27 | Ex9:c.1148G>A/p.G383D | Disease causing | Damaging | Damaging | Possibly damaging | Deleterious | Medium |
| 28 | Ex6:c.475C>T/p.R159W | Disease causing | Damaging | Damaging | Possibly damaging | Deleterious | High |
| 29 | Ex7:c.680A>G/p.N227S | Disease causing | Tolerated | Damaging | Benign | Deleterious | Low |
| 30 | Ex8:c.887G>A/p.R296Q | Disease causing | Damaging | Damaging | Possibly damaging | Deleterious | Medium |
| 31 | Ex4:c.242G>A/p.S81N | Disease causing | Damaging | Damaging | Possibly damaging | Deleterious | Medium |
| 32 | **Ex11:RecNcil** | | | | | | |
|  | Ex11:c.1448T>C/p.L483P | Disease causing | Damaging | Damaging | Possibly damaging | Deleterious | Medium |
|  | Ex11:c.1483G>C/p.A495P | Disease causing | Neutral | Damaging | Possibly damaging | Tolerated | Medium |
|  | Ex11:c.1497G>C/p.V460V | Disease causing | NA | NA | NA | NA | NA |
| 33 | **Ex6,7:Complex C** | | | | | | |
|  | Ex6:c.475C>T/p.R159W | Disease causing | Damaging | Damaging | Possibly damaging | Deleterious | High |
|  | Ex7:c.667T>C/p. W223R | Disease causing | Damaging | Damaging | Possibly damaging | Deleterious | Medium |
|  | Ex7:c.681T>G/p.N227K | Disease causing | Damaging | Damaging | Benign | Deleterious | Medium |
|  | Ex7:c.689T>G/p.V230G | Disease causing | Damaging | Damaging | Benign | Deleterious | Low |
|  | Ex7:c.703T>C/p.S235P | Disease causing | Damaging | Damaging | Benign | Neutral | Medium |
|  | Ex7:c.721G>A/p.G241R | Disease causing | Tolerated | Damaging | Possibly damaging | Deleterious | Medium |
|  | Ex7:c.754T>A/p.F252I | Disease causing | Damaging | Damaging | Benign | Deleterious | Medium |

Abbreviations: The Functional Analysis Through Hidden Markov Models (FATHMM), Not Applicable (NA), Polymorphism Phenotyping version 2 (PolyPhen-2), Protein Variation Effect Analyzer (PROVEAN), The Sorting Intolerant from Tolerant (SIFT)

† Impact of amino acid substitution on protein function
