# Supplementary material for: Gaucher disease: single gene molecular characterization of one-hundred Indian patients reveals novel variants and the most prevalent mutation
Source: BMC Med Genet. 2019 Feb 14;20:31. doi: 10.1186/s12881-019-0759-1 (PMC6376752; doi:10.1186/s12881-019-0759-1)
Supplement: Supplementary file 4 — ClinVar Accession ID of the variants generated in the given study. The variants identified through Sanger sequencing are reported in NCBI ClinVar database. The file provides accession ID and the links to an individual variant. (DOCX 14 kb) [file 12881_2019_759_MOESM4_ESM.docx]

The dataset generated and/or analyzed during the current study is available in the NCBI ClinVar repository. Following are the accession ID of the variants submitted to the ClinVar repository

1. **Variant c.1603C>T (p.R535C) in exon 12 of *GBA1* gene.**

ClinVar Accession ID: SCV000281966.1

[<https://www.ncbi.nlm.nih.gov/clinvar/variation/242383/>]

1. **Variant c.1459G>A (p.Ala487Thr) in exon 11 of *GBA1* gene.**

ClinVar Accession ID: SCV000282508.1

[<https://www.ncbi.nlm.nih.gov/clinvar/variation/236402/>]

1. **Variant c.167T>G (p.Val56Gly) in exon 4 of *GBA1* gene.**

ClinVar Accession ID: SCV000282509.2

[<https://www.ncbi.nlm.nih.gov/clinvar/variation/424819/>]

1. **Variant c.407C>T (p.Ser136Leu) in exon 5 of *GBA1* gene.**

ClinVar Accession ID: SCV000282507.2

[<https://www.ncbi.nlm.nih.gov/clinvar/variation/424818/>]

1. **Variant c.1448T>G (p.Leu483Arg) in exon 11 of *GBA1* gene.**

ClinVar Accession ID: SCV000786646.1

[<https://www.ncbi.nlm.nih.gov/clinvar/variation/93449/>]

1. **Variant c.1177C>G (p.Leu393Val) in exon 9 of *GBA1* gene.**

ClinVar Accession ID: SCV000282506.1

[<https://www.ncbi.nlm.nih.gov/clinvar/variation/236400/>]

1. **Variant c.415G>C (p.Ala139Pro) in exon 5 of *GBA1* gene.**

ClinVar Accession ID: SCV000282505.1

[[https://www.ncbi.nlm.nih.gov/clinvar/variation/236399/]](https://www.ncbi.nlm.nih.gov/clinvar/variation/236399/%5d)
